# How foreign aid and remittances affect poverty in MENA countries?

**DOI:** 10.1371/journal.pone.0261510

**Published:** 2022-01-19

**Authors:** Riyazuddin Khan, Zeeshan, Mohammad Imdadul Haque, Neha Gupta, Mohammad Rumzi Tausif, Isha Kaushik

**Affiliations:** 1 Department of Geography, Dr. Bhim Rao Ambedkar College, University of Delhi, New Delhi, India; 2 Department of Economics, Shaheed Bhagat Singh Evening College, University of Delhi, New Delhi, India; 3 Department of Economics, Faculty of Social Science, Aligarh Muslim University, Aligarh, India; 4 School of Business, Woxsen University, Telangana, India; 5 Prince Sattam bin Abdulaziz University, Al Kharj, Kingdom of Saudi Arabia; 6 Department of Geography, Post Graduate Government College, Chandigarh, India; Szechenyi Istvan University: Szechenyi Istvan Egyetem, HUNGARY

## Abstract

Foreign aid and remittances augment the income of economies. The present study examines the relationship between economic growth, poverty, inequality, remittances, and foreign aid in the Middle East and North Africa (MENA) countries using panel data methods from 1991 to 2019. The study focuses on the MENA region due to the rise in labor immigration and significant foreign aid. The empirical findings reveal that remittances, foreign aid, and economic growth play a significant role in bringing down the MENA region’s poverty levels. Besides, a rise in income share accruing to the lowest quintile is observed despite the negative income growth, which indicates that on average, the income increased more rapidly of the poor in comparison to the non-poor households. Thus, the study finds evidence supporting the hypothesis that remittances and foreign aid augment per capita income and income share in the MENA member countries.

## 1 Introduction

The challenge of poverty eradication is one of the most considerable challenges faced by the world. The Sustainable Development Goals (SDGs) put forth have replaced the Millennium Development Goals (MDGs), and poverty reduction remains central in the Post- 2015 Agenda [1, 2]. Hence, the growing need to find a solution to poverty eradication has heightened interest among researchers to find the main economic variables that can serve as a cure to poverty eradication. These economic variables differ across different regions, and particularly for low- and middle-income regions, foreign aid and remittance inflows have appeared to play an important role in achieving the aim of poverty alleviation [3–6].

It is well documented that economic growth is a function of various factors such as foreign capital, population growth, inflation, unemployment rate, etc. [7]. However, the MENA region has been significantly affected by its high unemployment rates, population growth, foreign aid, and government influence over its economic sectors [8]. The MENA region consists of both low and high-income countries. This region offers a strong contrast to much of the developed and developing economies in terms of the overall incidence of poverty and its poverty reduction trends. The poverty reduction in the region is attributed to the higher economic growth followed by the oil boom in the 1970s and early 1980s, which benefitted both the oil and non-oil exporters in the MENA region [9]. In terms of extreme poverty incidence, by the mid-1990s, MENA came after developing the eastern part of Europe and Central Asia. Only less than 2% of the population was living on less than US$ 1.00 per day during this time. However, one primary concern that has surfaced during the twentieth century concerns the sustainability of MENAs’ accomplishment in poverty reduction. After 1985, economic growth and poverty rate reduction have slowed down dramatically. Since then, the share of poor households living on US$2 per day has increased by the end of the 1990s [10]. Therefore, the importance of exogenous factors such as remittances, foreign aid, employment in the government sector, etc., needs significant attention to understand poverty dynamics in the MENA region.

One of the dynamic economic growth sources and development in emerging countries constitutes foreign aid as low foreign exchange revenues render insufficient social development and infrastructure projects. Some studies like [11] have found evidence favoring foreign aid’s positive impact in more significant growth in human capital, rapid accumulation of capital, and improved welfare for the recipient economies. The proponents believe in the positive effect of foreign aid as it helps the poor economies achieve higher economic growth levels by eliminating the difference between investment and saving. Foreign aid becomes vital for developing economies through better utilization of domestic resources and bridging the gap between savings and foreign exchange created due to lack of foreign currency. Usually, foreign aid is aimed to enhance economic welfare and support economic growth; however, foreign aid’s effectiveness on economic growth has been a substantial debate. Many empirical studies [11–16] have found mixed results.

Studies like [14, 17] advocated a positive impact of foreign aid on economic growth. While other studies, such as [18–20], concluded a negative impact due to misallocation of foreign aid, such as allocating the funds received for the non-developmental areas. [20] concluded a negative relationship between foreign aid and economic growth as foreign aid reduced labor supply and capital accumulation. They suggested that due to increased spending by the citizens, their leisure time increased, leading to a reduced labor supply. Furthermore, various other factors like political volatility, frequent policy changes, institutions’ ineffectiveness, and erroneous public sector priorities have reduced the effectiveness of foreign aid in eradicating poverty and promoting economic growth. Studies like [21] found that increasing foreign aid flows fostered the recipient economies’ economic growth with a conducive policy environment. In contrast, [22] suggested a negative relationship between aid and economic growth as increased aid flows reduced savings and increase consumption, leading to Dutch disease. The recipient country becomes vulnerable to the Dutch disease through which aid flows appreciate currency, thereby taxing the tradable export sector with damaging effects. This may also increase inflation, leading to a rise in non-tradable goods, reduction in exports, allocation of resources to non-tradable sectors, and an increase in imports. Therefore, it triggers more aid to finance the increased imports, resulting in aid dependency [22].

According to [5], remittances have grown to be an important source of foreign income in addition to foreign direct investments and official development assistance. Despite extensive literature, there is still no consensus on its impact on poverty reduction. Again, there are two thoughts of school–positive impact and negative impact on poverty reduction. Among the studies investigating the remittance-poverty nexus, some studies like [23–25] have posited a positive impact of remittances on poverty reduction. While other studies like [26] have found no evidence supporting an impact of remittances on poverty. Against this background, the current study attempts to examine the relationship between remittance inflows, foreign aid, income inequality, economic growth, and poverty reduction in the MENA region.

Hence, there are several ways in which the present study contributes to the literature. The majority of empirical studies on the impact of foreign aid and remittances on economic growth and poverty reduction have been carried out in the context of other countries or economic regions. Thus, there had been less focus on the MENA region, which is one of the major recipients of foreign aid. Secondly, the present study attempts to capture the underlying dynamics through the segregation of low-income and high-income MENA countries and examines the trends on a temporal basis, taking decade averages by partitioning the data. Thirdly, this study examines the impact of remittances, foreign aid, and economic growth on poverty reduction by considering two proxies, i.e., headcount ratio and poverty gap index, to establish the robustness of the results.

In this study, the impact of economic growth, remittances, and foreign aid on poverty and the poverty gaps examined in MENA countries. The empirical work is done in MENA countries employing econometrics methods using ordinary least square (OLS) regression and panel random-effect models, and system-GMM using panel data to support the results’ robustness. The data spans the period from 1991 to 2019 based on its availability from the World Development Indicator of the World Bank database. The study is divided into six parts, and the rest of this paper is organized as follows. Section 2 reviews the existing literature on the relationship between poverty, inequality, foreign aid, remittances, and economic growth. Section 3 focuses on a comparison of macro and survey-based poverty measurement approaches in MENA countries. The data source and adopted methodologies have been discussed in Section 4. While section 5 presents results, and section 6 concludes the study.

## 2 Nexus between different macroeconomic indicators

### 2.1 Poverty, inequality, and economic growth

The relationship between inequality and growth remains under considerable disagreement. The earlier studies on the relationship between growth and inequality mainly followed the Kuznets hypothesis [27, 28]. The hypothesis claimed an inverted U-shaped manner relationship. During the early phases of economic development, income inequality worsened (i.e., increased) until countries reached middle-income status, implying a positive association between the two. However, studies like [29–32] have suggested no significant relationship between them and rejected the Kuznets hypothesis. According to these studies, the income distributions generally remain stable over time as economic growth raises income for all society members.

Moreover, this is the reason why economic growth helps in poverty alleviation. The empirical finding that growth and inequality are unrelated underlies the results in a critical study by [33], whose analysis shows that Africa is well on the way to meeting the millennium development goal of reducing poverty by half. However, the extent of income inequality does play a significant role in the change of poverty levels caused by a change in economic growth. This view has been confirmed by [34], where the study examined 20 developing countries and found that even small changes in income distribution led to substantial changes in poverty incidence. The findings suggested that the fall in poverty levels was higher for a given economic growth rate when the income inequality declined.

[35] examined the poverty reduction performance in developing countries using available data from the World Bank. The study found that, except for the Eastern European and Central Asian (EECA) region, poverty measured at both $1 per day and $2 per day decreased for all the regions from 1981 to 2005. For measuring poverty, annualized growth rates of the headcount ratio based on $1 and $2 were used, while for measuring income inequality, the Gini coefficient was used. The study also found that a number of countries exhibited strong income growth; however, for these countries’ poverty reduction was low. For these countries, income inequality was found to be a significant mediating factor. For measuring the income inequality, the Gini coefficient has been employed by a multitude of the studies in various contexts. The Lorenz curve is a common graphical method of representing the degree of income inequality in a country and the Gini coefficient is the most used summary measure of economic inequality [36]. The application of Gini coefficient has expanded beyond the area of socioeconomics [37]. Areas such as human geography [38], public health [39] and many more have utilized the Gini index for measuring inequality. Along with the Gini coefficient, other measures of inequality have also been used such as Thiel’s index, Atkinson’s measures of income inequality, however, studies like [40] has reported that virtually all measures of income inequality are highly correlated. [41] investigated the impact of income distribution, measured by both functional and household approached, on social well-being for EU member states and Ukraine. The functional approach characterized the proportions of distribution of national income among the owners of the factors of production. While the household income distribution reflected the distribution of Gross National Income (GNI) between different households, regardless of their social standard and income sources. The study found that functional distribution of national income did not fully reflect the patterns in the income distribution. It indicated that due to conditions pertaining to high levels of “shadowing” of economic relations, official statistics become limited in reflecting the income distributions among population groups, as it only reflected inequality for the economy and population pertaining to the nonshadow economy. The Gini coefficient provides an integral criterion differentiating the income distributions among population groups and therefore, it serves as better indicator to understand the income inequality dynamics among countries. Finally, the study concluded that fair income distribution, on both functional and household level, positively impacted the possibilities of achievement of social and economic well-being.

Thus, it can be observed that the level of income inequality and economic growth in a region plays an important role in attaining poverty eradication.

### 2.2 Foreign aid and economic growth

The literature on the impact of foreign aid has adopted a wide range of approaches and reached radically different conclusions. Let us take an example of two relevant studies that illustrate the diversity of the results. [15] found little robust evidence favoring any relationship between foreign aid, economic growth, and poverty reduction in a given country. The study did not find any evidence about the better effectiveness of foreign aid in conducive policy environments or geographical environments or that certain aid forms work better or are more helpful. In contrast, using a similar approach as [15, 42] provided strong evidence supporting the idea that aid impacts growth positively as it stimulates aggregate investment and increases productivity. What explains such different conclusions? Much of the work in this area has been, at least in part, a response to a very influential study by [21], which argued that aid did have an impact on economic growth and poverty reduction but only in a good policy environment. A conclusion directly rejected by [15]. In this section, the study intends to focus not on the details of many studies on this topic but on the underlying specification issues that will be important for establishing the conclusions the study wants to draw.

Whether foreign aid helps reduce poverty and its gaps in MENA countries or not is still subjected to further exploration. Studies advocating the negative relationship between economic growth and foreign aid claim that due to the increased aid inflows, the consumption increases, and the domestic savings decrease, which hinders the recipient countries’ economic growth. The negative relation between economic growth and foreign aid may also result from various other factors like economic policy interventions, business cycles, government intervention, and dependability of foreign aid inflows in the poor beneficiary countries. [43] examined the effect of foreign aid on savings, investment, and growth for 39 sub-Saharan African countries from 1986–92 and found that foreign aid improved economic growth for countries with prolonged imbalances or negative per capita growth. The study also indicated that foreign aid impacted the domestic savings negatively, but the effect was concentrated for the countries with negative per capital growth. Besides, a significant role of sound economic policies in foreign aid effectiveness in promoting economic development has also been suggested. Other studies which have advocated a positive impact of foreign aid on economic growth are [5, 3, 21, 44].

[15] examined all the developing economies that had gotten aid during the post-war period and studied foreign aid’s effects on growth in both panel and cross-sectional settings. The study found very slight robust evidence of a positive or a negative relationship between the aid inflows and the country’s economic growth. Furthermore, the study did not provide evidence that aid is more effective in conducive policy environments or works better for specific geographical regions. [4] examined 111 countries from 1970 to 2005 to study the effectiveness of foreign aid on income distribution and poverty and found no statistically significant effect of aid on inequality and poverty reduction. Furthermore, the study failed to detect any foreign aid contribution in promoting growth, even when the institutional quality is considered. This was in line with the study by [45], who also identified foreign aid’s failure to promote economic growth despite good institutions.

[8] used simultaneous equation system to investigate foreign aid’s effect on economic growth and domestic savings and found a negative relationship between them for eight MENA countries from 1977 to 2013. The study also suggested a negative impact of foreign aid on the recipient countries’ domestic savings, hindering economic growth in a lousy policy environment. [13] empirically investigated foreign aid’s role on domestic savings and economic growth in SAARC member countries from 1980 to 2008 using a simultaneous equation system. The study inferred a positive and significant impact of foreign aid on the five nations’ growth; however, a negative relationship was observed between foreign aid and domestic savings. The study suggested taking the net effect as the positive effect of foreign aid might get offset by the negative impact of aid on domestic savings. [16] analyzed the direct and indirect effects of aid flows on developing countries’ economic growth for a sample of recipient countries from 1960 to 1999. The study focused on the disbursal aspect of the funds and found that aid had a direct negative effect on growth, did not increase investment, and had a significant positive impact on government consumption. They argued that due to rent-seeking activities induced by the more comfortable fund flow resources, the funds flow to non-productive activities, affecting the investment. Besides, this favouring of one group over the others due to rent-seeking behaviour leads to an inefficient increase in government consumption and results in a deleterious impact on other groups.

[46] studied three southeast Asian economies, namely Thailand, Indonesia, and the Philippines, and empirically tested foreign aid’s effectiveness in promoting economic growth during 1970–2000. The study found no evidence that foreign aid had an insignificant impact on economic growth and domestic savings. Besides, [47] indicated that aid has a decreasing return, i.e., each additional dollar of aid has a smaller (positive) impact on growth than the preceding dollar. Therefore, when aid did not adversely impact local investment and domestic savings, the relationship between aid and growth is positive. Finally, aid has been the main driver in contributing to economic growth and promoting development in poverty reduction. Still, such goals remain dependent on various other factors, including proper allocation of aid to economic sectors, types of government expenditure, geographical regions, and approach towards poverty.

[48] assessed the impact of foreign aid on poverty reduction in Nigeria using an ARDL and error correction model (ECM) from 1981 to 2014. The study used household consumption expenditure as a proxy for poverty reduction. Although a positive influence was suggested of foreign aid on poverty reduction, the impact was found out to be insignificant. [49] analyzed the impact of foreign direct investment, trade, and foreign aid on poverty alleviation for Sub-Saharan African (SSA) countries. The study used data from 1990–2017 and employed the Feasible Generalised Least Square (FGLS) technique. To measure poverty, the human development index was used. The results indicated a negative impact of foreign aid on poverty reduction. [6] investigated the causal relationships between foreign aid, economic growth, and poverty for 82 developing countries using panel vector error correction model (VECM) Granger causality test. The study used headcount rate as the measure of poverty and GDP per capita for measuring economic growth. The short and long-run causality linkages suggested that aid was generally allocated to developing countries with high poverty levels and a lower GDP per capita. Further, it was also suggested that foreign aid is not a long-term solution for poverty alleviation.

### 2.3 Remittances and economic growth

Remittances constitute a significant source of financial flows for developing economies, and their effect on economic growth is being debated for a long. There is extensive literature examining the impact of remittances on economic growth and poverty alleviation, and the following section attempts to highlight some of them. Studies like [23] provide evidence on the positive contribution of remittances to economic growth through positively influencing savings, consumption, or investment. [50] examined a sample of over 100 countries for the period 1975–2002 and found that remittances accelerated the economic growth and made up for the lack of proper financial markets. The results suggested that remittances helped in reducing credit constraints for the poor and improved the allocation of capital thus, enhancing financial development and, in turn, promoting economic growth. [51] analyzed a sample of 39 developing countries to investigate the effect of remittances on economic growth spanning from 1980–2004 using a standard growth model. The results indicated a positive impact of remittances on economic growth. [52] employed panel cointegration to examine the effects and the channels through which remittances affected the economic growth by following the growth model [7]. The study used an unbalanced panel of 40 countries from 1970 to 2006 and found the remittances influencing economic growth directly and indirectly. The study estimated that doubling the remittances from 5 percentage points to 10 percentage points led to an increase in growth by approximately 0.3 percentage points in the sample under study.

In contrast, studies like [53] have advocated an adverse effect of remittances on economic growth due to the movement of resources from the tradable sector to non-tradable sectors and appreciation in the real exchange rate. The study by [54] also echoed similar findings while analyzing 84 countries during 1970–2004 and confirmed a negative impact of remittances on economic growth. [55] also highlighted a negative relationship between the two and applied Dutch Disease theory to support the view. [56] examined a panel of 113 countries and indicated a negative effect of remittances on economic growth during 1970–1998. The study presented uniqueness in bringing in the problem of moral hazard and the compensatory nature of the remittances, which resulted in its negative impact on the economy.

Regarding the effect of remittances on poverty reduction, a study by [57] found a small positive impact of remittances on growth but suggested a significant positive influence on poverty reduction while examining a panel of selected Asian and Pacific countries during 1993–2000. Similarly, a study by [50] analyzed a sample of 101 developing countries and found a significant positive impact of increasing remittances on reducing poverty. However, no effect was found on economic growth. [23] used time-series cointegration to study the relationship between remittances and poverty reduction for Ghana and posited the evidence supporting the strong effect of remittances on poverty reduction. [58] examined more than 20 Asian countries from 1988 to 2007 to analyze the impact of remittances on economic growth and poverty. The results revealed a positive effect of remittances on growth and a negligible impact on poverty; however, remittances reduced the poverty gap and enhanced poverty depth. [59] examined the implications of remittances on poverty reduction for 44 developing countries from 2006 to 2014 and found strong evidence supporting low poverty levels in countries with high remittances flow. [60] assessed the impact of remittances on economic growth and poverty reduction for selected 10 Commonwealth of Independent States (CIS) countries using panel data from 1997to 2016. Three poverty measures, namely poverty headcount, poverty gap, and squared poverty (poverty severity), were used to proxy for poverty. The study revealed two important channels through which remittances were found to affect poverty among the CIS countries. Remittances were found to significantly reduce poverty by smoothening the consumption levels and increment in income levels. [2] studied the impact of remittances on poverty in a single country context, i.e., Botswana. The study employed time-series data from 1980 to 2017 and used two poverty measures, i.e., household consumption expenditure and infant mortality rate. Autoregressive distributed lag (ARDL) approach was used for the short term and long-term analysis. The findings revealed that when infant mortality rate was used as proxy for poverty, remittances were found to reduce poverty in both short and long term. However, no impact on poverty levels was observed when poverty was measured by household consumption expenditure.

## 3 A comparison of macro and survey-based poverty measurement approach in MENA countries

There are two approaches to poverty measurement, namely: Macro based and Survey based. The macro-based approach employs income, while household surveys use a consumption-oriented approach to measure poverty levels. One of the significant constraints faced by the researchers who try to identify the poverty in the MENA region is the absence of comparable and unavailability of the household surveys. This creates extreme difficulty in the identification of the extent of poverty in the region under study. As reported by [61], data on poverty and income inequality is available only for 9 out of 15 MENA countries. Only four member countries have openly accessible nationally representative household budget surveys limited to 1 year only.

The macro-based approach utilizes international and national poverty lines to measure the level of poverty. Both national and international poverty lines have their advantages and disadvantages. However, international poverty lines are more suitable for cross-country and temporal comparisons of poverty levels in different regions [62]. Nevertheless, poverty lines defined internationally (US$ 1.90 per person per day) are arbitrary as one size does not fit all, i.e., setting US$ 1.90 per person/day for all the developing countries is ambiguous. Therefore, while examining poverty dynamics at the national level, the poverty lines prove acceptable/suitable as they are close to the real expenditure of food and non-food items in different countries. Even though the study neglects to utilize the national poverty lines as many developing countries (including MENA members) report sparse data on nutrition, which makes it is extremely difficult to accurately set "nutritional requirements" and specify the cost of "non-food needs" precisely.

Survey-based and macro-based data tell a very different story when compared. For example, when measuring poverty in the African population, [33] found a fall of 5 percentage points using macro data, while an increase (from 49 to 54 percentage points) in the headcount poverty rate using the survey-based data during the period from 1993 to 2001. However, over the period from 2001 to 2009, both datasets reported the same direction. However, considering the whole period, the two sources of data differed in the level of poverty. In 2009, the survey-based data reported a data figure of 47 percentage points, while in 2006, the macro data reported a figure of about 33 percentage points. This proves the existence of a considerate deviation between the figures reported by the two datasets.

Now the question which arises is which dataset is correct? Both of them have their fair share of problems, and hence, "neither of them" is the answer. Many reasons render both approaches leading to very different views on poverty levels and associated temporal trends. For example, the macro approach considers the measurement and modelling of national income at the aggregate level. Doing it within a country leads to many problems, especially in countries with extreme poverty. This is due to unmeasured or under-measured economic activity occurring in small–scale informal markets, which is extremely difficult to be accounted for by national account statisticians. At the same time, comparison across countries becomes extremely problematic as well. As indicated by [63], factors pertaining to the informal sector (shadow economy) can distort the perceptions of the true state of an economy. Income and employment in the informal sector can mislead or misrepresent the true picture related to the formation, distribution and redistribution of income in the form of social transfers. And, it’s a well-known fact among the research community that it is impossible to eradicate the informal sector completely.

Another reason why macro data differs from survey-based data is the indicator used to measure poverty. As mentioned earlier, the internationally comparable income numbers can deviate from the consumption numbers that emerge from the household surveys. Both income and consumption follow different trajectories within a country. For example, in the short term, due to higher investment, the national incomes rise, but if the returns on the investment are low, then there may be no long-run gains in consumption. Secondly, the divergence between the two may occur due to primary commodities-oriented exports of many poor developing countries, as in Africa. Therefore, the nature of the product becomes a critical factor whenever a boom occurs. For example, due to the oil boom, much of the gains from an increase in income would be captured by the government. In contrast, for an agricultural boom, where income accrual occurs at small-scale farmers’ level, the results might paint a different picture.

### 3.1 Why has macro data approach been chosen?

How does aid affect poverty? How do remittances affect poverty? These questions have been prominent in many development debates, in which opposing views have often relied heavily on macro data and panel methods. There are two main reasons why panel models have been more prominent in macro than micro-work in development.

The first is that the data demands are considerable and the number of micro data sets for emerging countries, while far larger than it was 20 years ago, remains small. In contrast, the number of macro data sets, time being what it is, has been growing continuously. Also, many organizations produce macro data that is designed to be comparable across countries. Such sources now included data on financial aid received for development works and remittances received from non-residents. The comprehensive economic data world comprises the World Bank Development Indicators, the Penn World Tables, and the [64, 65] data on education. These, and many other data sets, have led to an explosion of empirical work on macro topics, and much of this work uses panel estimators.

The second reason is that governments want answers to questions that macro data can answer. High on the list of the questions in development that public-sector bodies want to be answered is whether it promotes growth and reduces poverty and, if it does not, what does? The relevant literature shows almost as many answers to those questions as studies on the subject. Clearly, a first step to answering those questions is to understand why so many different answers have been possible using very similar data, very similar specifications, and similar estimators. If empirical work is successful, the study would expect a narrowing of the possible answers to the questions posed, but that has not been happening.

Also, the limited data on national household budget surveys pose a significant limitation, especially for smaller population developing countries that seldom conduct such surveys. Furthermore, in the countries where such data is available, it is only compiled for one year. In this aspect, a panel data set was collected for some countries by including additional information on income, poverty, remittances, and aid. Still, due to the limited period and data unavailability, some dataset was dropped. The panel approach resulted in an unbalanced panel allotting high weights to few observations on the remittances variable thus, abandoning the survey-based approach in favor of a macro data approach.

## 4 Data source and econometric models

This study uses balanced panel data of 21 MENA countries from 1991 to 2019 from the World Bank Database. The variables considered for the study are poverty headcount ratio as a proxy for poverty, per capita GDP as a measure of economic growth, Gini index as a measure of income inequality, foreign aid, and remittances as a percentage of the total GDP. According to [66], the headcount ratio is the most recognized indicator of poverty. It simply measures the proportion of the population considered to earn an income less than the standard required for basic needs. Even for Sustainable Development Goals (SDGs), the headcount ratio is the primary yardstick for measuring monetary and multidimensional poverty [1].

For the present study, GDP per capita has been used for measuring the economic growth of the MENA region, as with most previous literature on economic growth. It is defined as a measure of a country’s economic output accounted for its number of people. Mathematically, it is calculated by taking a ratio of the country’s GDP divided by its total population. Unlike GDP growth, GDP per capita is a better measure of the standard of living in a specific country. Growth in the production of goods and services is a primary determinant of how the economy is performing. GDP per capita shows the extent to which the total production of a country is shared among its population. Further, this indicator is helpful when the comparison among the countries is to be carried out.

Although GDP per capita is the most widely used measure of living standards, it is also a target indicator for policy-driven analysis. However, it fails to consider the people’s well-being due to persisting income inequalities among the population. Therefore, to take care of the impact of income equalities among the MENA region, the Gini coefficient has been used to measure income inequality which is the most popular measure. The advantage of the Gini index is that inequality of the entire income distribution can be summarized using a single statistic between 0 and 1. This characteristic allows for comparison among the countries with different population sizes [67]. Additionally, the data on the Gini index is regularly updated and reported by different countries and is easy to access.

The empirical models are based on the poverty reduction equation in terms of headcount ratio and the poverty gap equation as follows.


$$HCR_{\mathrm{it}}=\beta_{o}\log EG_{\mathrm{it}}+\beta_{1}GI_{\mathrm{it}}+\beta_{2}\log NA_{\mathrm{it}}+\beta_{3}R_{\mathrm{it}}+{ϴ}_{t}+C_{i}+U_{\mathrm{it}}$$ (1)

$$PG_{\mathrm{it}}=\beta_{o}\log EG_{\mathrm{it}}+\beta_{1}GI_{\mathrm{it}}+\beta_{2}\log NA_{\mathrm{it}}+\beta_{3}R_{\mathrm{it}}+{ϴ}_{t}+C_{i}+U_{\mathrm{it}}$$ (2)

Where *HCR*_it_ is the poverty headcount ratio at $1.90 a day, *logEG*_it_ is the value of the natural log of per capita GDP, *GI*_it_ refers to the Gini index (a measure of income inequality), Net aid (*logNA*_it_) and remittances (*R*_it_) are in the percentages of the total GDP. ϴ_t_ represents time fixed effects, and C_i_ is country fixed effects. All monetary values are expressed in real PPP US$ 2011 and β_0_, β_1_, β_2_, and β_3_ are parameters to be estimated, and U_it_ is the error term. However, in Eq (2), *PG*_it_ refers to the poverty gap. The poverty gap indicator produced by the World Development Research Group measures the poverty by considering the household per capita income and consumption. The poverty gap shows the average shortfall of the total population from the poverty line, which is the minimum level of income needed for survival through securing the basic necessities.

The key features of Eqs (1) and (2) are (i) that the model is explicitly dynamic; (ii) factor inputs are not included; (iii) the variables that are to be investigated as potential determinants of poverty may operate either through factor accumulation or through total factor productivity (TFP). The study focuses on GDP per capita, aid, and remittances. The coefficient on aid has been the focus of the discussion, and GDP per capita and remittances are two elements that enter the policy measure used by [21].

We also checked the robustness using a system-GMM approach to control serial correlation and cross-sectional dependence. We did the two-step system-GMM estimation for both our models. Our random effect estimates may have biased estimates owing to several characteristics. Foremost, there is a potential endogeneity problem. There could be a correlation between regressors and the error terms and the omitted variables bias, such as the absence of relevant lagged variables in the model. Next, the random effects may result in biased OLS estimates. Lastly, the error term may have heteroscedasticity and serial correlation despite being uncorrelated across countries. Therefore, estimation using a static panel random effect model may be biased. The system GMM estimator solves this problem by using past differences as instruments for levels; it depends on an initial condition restriction which may be highly suspect with macro data. Hence, to control all these model issues with the random-effect model specifications, we have done the system-GMM estimation and included the lagged variable of the outcome variable to control the serial correlation and instrumental variables to deal with the endogeneity issues among regressors. The use of the system-GMM technique as a potential approach to address the endogeneity problem is well supported by the existing literature [26, 52, 68].

## 5 Results

The estimates of poverty, inequality, and economic growth for different regions, including the MENA region during 2000–2019, are presented in Table 1. The estimates are presented as decade averages in order to expedite temporal comparison. The international poverty lines are defined based on the estimations of $1.90 per person/day in 2011 following purchasing power parity (PPP). The PPP values are determined by valuing a bundle of goods in each country/region and matching the bundle’s local cost with the U.S. dollar cost of the same bundle. In this study, the PPP exchange rates are used to make the $1.90 threshold roughly the same in all countries.

**Table 1:** Poverty, inequality and growth data by region.

Region Name	Poverty Headcount Ratio ($ 1.90/person/day)			Gini Index			Income share held by lowest 20 percent			GDP per capita, PPP (constant 2011 international $)		
	Average		Percentage Change	Average		Percentage Change	Average		Percentage Change	Average		Percentage Change
	2000–2009	2010–2019		2000–2009	2010–2019		2000–2009	2010–2019		2000–2009	2010–2019	
East Asia and Pacific	14.83	7.76	-47.72	38.36	37.44	-2.39	6.61	6.91	4.56	16063	20819	29.61
Europe and Central Asia	3.57	1.10	-69.10	32.18	31.75	-1.34	7.84	7.79	-0.66	25182	28242	12.15
Latin America and Caribbean	9.37	5.33	-43.06	51.43	47.11	-8.40	3.60	4.41	22.48	15792	17085	8.19
Middle East and North Africa	4.20	3.54	-15.76	36.05	34.61	-3.99	7.23	7.47	3.33	30612	28630	-6.48
North America	0.55	0.71	28.79	37.13	37.38	0.67	6.17	5.98	-2.97	47292	49482	4.63
South Asia	22.34	10.53	-52.88	37.48	34.93	-6.81	7.58	8.03	5.92	4206	6041	43.63
Sub-Saharan Africa	46.77	38.09	-18.54	43.87	43.19	-1.56	5.68	5.74	1.08	4118	4909	19.20
World	11.93	7.48	-37.31	38.88	36.92	-5.05	6.48	6.77	4.47	15980	18063	13.04

Source: Authors’ estimations using data from World Development Indicators

MENA region is unique as this was the developing region with the lowest poverty incidence when the transitional economies of North America, followed by Europe and Central Asia, started with a lower prevalence of poverty. Table 1 reveals some of the key findings, which are discussed in subsequent sections. First, as determined by the $1.90 per person/day standard, poverty is lower than in the other developing regions. All the regions except North America have experienced a decrease in their poverty levels, including MENA, where poverty has decreased by about 16% (as measured by headcount poverty ratio) during the last two decades. For North America, the headcount ratio is below 1%, followed by Europe and Central Asia, and MENA regions. Second, in terms of the Gini coefficient, MENA stands out as a bottom inequality with low Gini coefficients comparable to East Asia and Pacific, Europe and Central Asia, North America, and South Asia. It is also comparable with world measures.

Compared to the Gini coefficient, the MENA region has one of the equal income distributions globally, with the Gini coefficient being equal to 34.61 percent compared to the world’s Gini being equivalent to 36.92. Third, the reduction in income inequality is mainly due to the increasing share of income among the bottom quintile, which grew over 3 percent between the 2000s and the 2010s. Despite the low growth rates, the region has accomplished low poverty rates with nearly equal income distribution. In the last two decades, only the MENA region observed a negative rate of per capita GDP growth with a decline of 6.5 percent. Still, the region has been able to sustain low poverty levels. This pattern of negative growth of per capita GDP, increased share of income among the poorest quintile, reductions in inequality, and a low and, to some extent, steady poverty headcount index is exceptional to the MENA region. The results present a contrast to the general relationship found in the literature, which states increase in economic growth helps in poverty alleviation and, at the same time, has little impact on income inequality. The findings are in line with the econometric study examining 50 developing countries by [69]. The results suggested a positive effect of economic growth on poverty reduction as the poverty elasticity for growth is 2.59. It indicates that, on average, a decrease of 25.9% in the proportion of the people living in poverty, set at $1.00 per person per day, is expected when the growth increased by 10%. Further, the study also indicated an insignificant impact of economic growth on income distribution, i.e., the economic growth did not affect the income inequality, and it may fall or rise with economic growth.

As evidenced, MENAs’ accomplishment in cutting the poverty rate after negative economic growth needs to be considered. Measures of poverty include headcount ratio, and any change in headcount poverty would result in a change in income (or consumption) and its distribution among income classes. Thus, increasing income per capita will translate into a reduction in poverty for any given income distribution. However, this does not mean that all the income groups are affected equally because average growth does not necessarily translate into the growth of the poor’s incomes. According to [70], growth, which results in progressive distributional change, has a more significant impact on poverty reduction than growth, rendering the distribution unchanged. On the other hand, growth, which results in regressive distributional change, can offset the benefits of growth to the poor. A decline in income inequality directly affects poverty reduction at any growth rate and further increases the impact of future growth on poverty eradication.

MENA’s success in achieving minimal poverty rates by the 1980s and sustaining into the 1990s has been attributed to economic growth before the 1980s. The region’s oil-fired economic boom of 1975 to 1985 helped MENA reduce its poverty levels as the per capita GDP grew at 4.5% per year [69]. The rapid economic growth had an influential impact as $1.00 per day poverty declined by nearly two-thirds in the region between 1975–79 and 1985–89. As mentioned in the above section, the fall in income inequality during this decade increased the impact of prolonged future growth on reducing poverty levels during the 1990s.

As [51] has pointed out, it is necessary to remember that falls in poverty rates do not necessarily imply falls in the number of the poor. Therefore, two poverty measures, namely headcount ratio and poverty gap, are studied and presented in Table 2. The first, the headcount ratio, calculates the percent of the population living under US$ 1.90, US$ 3.20, and US$ 5.40 per person per day. However, it does not consider the gap or the amount by which the expenditure (income) by which the poor fall short of the set poverty standard. Hence, to cover this limitation, the poverty gap index estimates are also presented in Table 2 shows the poverty gap index. This helps in determining how far the incomes of the poor lie from the set poverty line. For example, a poverty gap of 10% suggests that the average poor person’s expenditure (income) falls short by 10% from the poverty line and has income equal to 90% of the poverty line.

**Table 2:** Growth, distribution, and poverty reduction in developing MENA, 1990–2019.

	High-Income MENA Countries			Low-Income MENA Countries		
	2000–2009	2010–2019	Relative Change	2000–2009	2010–2019	Relative Change
Poverty HCR at $1.90 a day	0.41	0.20	-51.72	3.26	4.28	31.24
Poverty HCR at $3.20 a day	0.63	0.44	-30.00	15.24	14.03	-7.94
Poverty HCR at $5.50 a day	2.30	1.68	-26.96	41.41	37.36	-9.77
Poverty gap at $1.90 a day	0.16	0.08	-49.09	0.71	1.32	86.58
Poverty gap at $3.20 a day	0.29	0.18	-37.00	3.92	4.30	9.59
Poverty gap at $5.50 a day	0.74	0.51	-31.35	14.24	13.31	-6.55

Source: Authors’ estimations using data from World Development Indicators

Note: High Income MENA countries refer to Bahrain; Israel; Kuwait; Malta; Oman; Qatar; Saudi Arabia; and the United Arab Emirates

Low-Income MENA Countries refer to Algeria; Djibouti; Egypt; Iran; Iraq; Jordan; Lebanon; Libya; Morocco; Syria; Tunisia; West Bank and Gaza; and Yemen

Table 2 compares average poverty for different measures of 2000–2009 with 2010–2019 between high-income MENA countries and low-income MENA countries. The low-income countries together comprise 61.9 percent of the total distribution of 13 MENA countries. The high-income countries comprise eight countries with coverage of 38.10 percent of the distribution. All three measures cover impoverished households. The average in Table 2 shows a similar pattern for all three poverty measures for high-income MENA countries, in that there is a decrease in poverty from 2000–2009 to 2010–2019. In contrast, there is an increment in poverty measures of US$1.90 for low-income MENA countries, then a decline in poverty measures of US$3.20 and US$5.50 from 2000 to 2019.

Table 3 presents ten estimated versions of Eqs (1) and (2) together. Columns (1) to (5) use data averaged poverty headcount ratio measure at US$1.90, from 1990 to 2019, which yields n = 1280 observations for the first four models (columns 1 to 4) and 780 for the fifth model (column 5) because a dummy of low-income MENA countries has been applied. Similarly, in columns (6) to (10), the results using the averaged poverty gap at US$1.90 as a dependent variable are reported. In Columns (6) and (10), the study estimates the specification of Columns (1) to (5), which yields the same observations across the MENA countries. The random effect results and the estimates of fixed effects, and the Hausman test have been given in the S1 Appendix. The coefficient estimates suggest that the poverty elasticity is negative for per capita GDP (income), i.e., an increase in growth leads to a decline in poverty.

**Table 3:** Elasticity of poverty concerning foreign aid and remittances (random effect).

	Dependent variable = poverty headcount at $1.90/person/day					Dependent variable = poverty gap at $1.90/person/day				
	(1)	(2)	(3)	(4)	(5)	(6)	(7)	(8)	(9)	(10)
ln GDP per capita	-11.87***	-11.01***	-17.77***	-19.62***	-19.50***	-4.553***	-4.046***	-7.071***	-7.556***	-7.511***
	(-39.30)	(-33.04)	(-34.25)	(-42.95)	(-43.14)	(-31.97)	(-25.87)	(-28.37)	(-32.86)	(-32.80)
Gini Index		0.128***	0.454***	0.315***	0.290***		0.0940***	0.245***	0.190***	0.181***
		(3.32)	(9.95)	(8.24)	(7.57)		(5.21)	(11.19)	(9.87)	(9.33)
ln net aid received			-0.102	-0.0587	-0.0300			-0.0454	-0.0262	-0.0158
			(-1.09)	(-0.77)	(-0.40)			(-1.01)	(-0.68)	(-0.41)
Remittances				-0.834***	-0.831***				-0.307***	-0.305***
				(-17.25)	(-17.39)				(-12.60)	(-12.63)
MENA excluding LIC					-5.778***					-2.090**
					(-4.36)					(-3.11)
Constant	128.6***	115.0***	165.9***	193.2***	192.9***	49.25***	40.44***	62.98***	71.22***	71.11***
	(41.60)	(27.81)	(28.27)	(36.76)	(37.17)	(34.12)	(20.84)	(22.34)	(26.92)	(27.04)
R-square	0.5648	0.5415	0.6349	0.7388	0.7456	0.4586	0.4421	0.5567	0.6348	0.6397
Observations	1260	1260	1260	1260	780	1260	1260	1260	1260	780

Note: Significance level of the difference

* p<0.05

** p<0.01

*** p<0.001

Source: Authors’ calculations, using data from World Development Indicators

Robust standard errors clustered at the country-level in parentheses.

Similarly, the Gini coefficient estimates are positive, which is expected as a rise in Gini coefficient means an increase in income inequality, leads to an increase in poverty level. The estimates and their magnitudes follow the literature [69] and are statistically significant for all 10 cases. For example, in all the models from Columns (1) to (10), the sign of GDP per capita is negative, highly significant, and consistent, proving the robustness of the estimates. Recent studies like [71, 72] have advocated for the inclusion of the initial income inequality variable as a control variable. The results have been inferred as poverty elasticities concerning remittances or foreign aid. In Columns (3) to (5) and Columns (8) to (10), the parameter estimate on aid is negative but not significantly different from zero. This indicates that foreign aid either does not matter or drives growth. Therefore, both the conclusions can be generated from the same and the same estimator.

In this study, remittances are included as an explanatory variable as the study’s objective is to examine whether foreign aid enhances domestic savings in increasing gross investment in the economy. Foreign aid includes all the government aid aimed at promoting economic development and the welfare of the recipient economies. However, sometimes the recipient governments pursue non-developmental agendas and misallocate the funds to less important sectors than allocating them to the needful sectors. Our interest lies in whether foreign aid helps domestic savings in increasing gross investment in the economy. Therefore, remittances have been included as an explanatory variable for carrying out the analysis. In this study, panel data evidence shows that international migration/remittances and foreign aid are two key factors that have helped MENA countries maintain low poverty rates despite stagnant economic growth. MENA countries have received a significant amount of foreign aid that has been used by the government in various public sector activities (include government work and work for public sector enterprises) to keep people working and eventually come out of the poverty trap. In addition, beginning of the 1970s, the global migration to the European and Persian Gulf countries has also climbed, which has helped the poor throughout the MENA region boost their incomes. During the oil-fired economic boom in the late 1970s, the oil prices rose significantly, leading poor people from countries like Egypt and Jordan to Persian Gulf countries like Iraq, Kuwait, and Saudi Arabia, looking for high-compensating occupations in a diversity of labor-intensive sectors. Simultaneously, the migration to Western Europe took place from countries like Algeria, Morocco, and Tunisia for the same reason. This resulted in a substantial effect of international remittances on the poverty levels across the MENA region. Therefore, foreign aid and international remittances on the poverty levels are examined in the following sections.

Table 3 also introduces the results examining the effect of remittances on poverty. For the complete sample of 21 MENA countries, the remittances are observed to have a statistically significant and negative influence on poverty (Column (4), (5), and Column (9), (10)). This indicates that a higher remittance negatively impacts both the poverty headcount ($1.90 person/day) and the poverty gap after adjusting the per capita GDP and income distribution. However, these numbers undervalue the amounts of remittances running back to these countries. This is mainly since they include only remittances that return via formal banking channels. The unofficial and informal means of remittances do not get covered as many migrants pay their money back home through informal channels because of the lack of trust in the banks [9, 10]. Unfortunately, data for those informal and unofficial means cannot be extracted; hence, the study relies on the available data that gives fair inference of the dynamics of remittances in MENA countries.

In Table 3, Columns (5) and (10) present some interesting results. When a shift dummy variable is applied for the MENA region, excluding LIC observations demonstrates a significant harmful effect. Besides, the Gini coefficient decreases for both the poverty measures indicating the robustness of the analysis and the overall explanatory power of the regression also rises. This suggests that adjusting for the level of income and income inequality, poverty in the MENA high-income countries is significantly below the poverty measure of $1.90. Foreign aid and international remittances help in poverty alleviation.

Table 4 shows the results of applying the system GMM using the exact specification as in Eqs (1) and (2). The problem of weak instruments can result in radical changes when different instrument sets are used. To keep comparability across the specifications, both the regression reported in Table 4 use two or three-period lags of all the regressors. It is usually suggested to experiment with different lag structures when using these estimators (citations). Focusing on column (1), we note that at the 1 percent significance level, the GDP per capita, foreign aid, and remittances are not positive and highly significant, suggesting that a 1 percentage point increase of the GDP per capita, foreign aid, and remittances will, in the long run, decrease poverty headcount ratio by 19.77, 0.23, and 0.66 percent respectively that seems a very substantial effect. Similarly, column (2) results in Table 4 can be interpreted and can be compared with Table 3.

**Table 4:** Dynamic panel estimates using a system-GMM approach.

	Poverty Headcount	Poverty Gap
ln GDP per capita	-19.7703***	-7.1670***
	(0.2398)	(0.0721)
Gini Index	0.4946***	0.2771***
	(0.0108)	(0.0031)
ln net aid received	-0.2346***	-0.0945***
	(0.0397)	(0.0139)
Remittances	-0.6481***	-0.0648***
	(0.0071)	(0.0019)
MENA excluding LIC	-9.3362	-6.2662
	(7.8515)	(7.2648)
Constant	188.3665***	62.4713***
	(3.1356)	(0.7975)
No. of Observation	715	715
Hansen	39.4195	40.8120
Hansen (p-value)	0.9979	0.9964
ar2	0.6633	0.9737
ar2 (p-value)	0.5071	0.3302

Note: Significance level of the difference

* p<0.05

** p<0.01

*** p<0.001

Source: Authors’ calculations, using data from World Development Indicators

Robust standard errors clustered at the country-level in parentheses.

Hence, the results of the system GMM are robust to the random effect model. The increase in overall GDP per capita in the region is negatively associated with poverty headcount and the poverty gap in the middle east region. This is in line with the results finding by [26, 52, 68]. Similarly, we see the benefit of foreign aid received and the remittances in the region and supported by the studies [2, 6, 49, 60]. In poor countries in the MENA region, we see more poverty gap and headcount ratio, highlighting the fact that the non-poor countries did a great job in reducing inequality and poverty in the region. This also indicates that poor countries in the region need to investigate these parameters to strengthen the development in the region. Since they lack foreign aids and remittances, it needs more investments in remittances and foreign aid to reduce poverty in the region overall.

## 6 Conclusion

The debate concerning foreign aid and international remittances in reducing poverty in the macro literature has been concerned with two major issues. The first has been how aid and remittances can be instrumented to find a casual effect. The second has been the role of policy and whether aid and remittances are effective only in suitable policy environments.

The results are best understood as describing how foreign aid and remittances impact poverty and its depth over the period covered by the data, 1990 to 2019. The estimates imply that remittances can be shown to have impacted significantly on both the poverty and the poverty gap. However, the relationship between foreign aid and poverty reduction is negative but not statistically significant, maybe because of conditioning on the time-invariant growth rates across countries. The regressions imply, are much lower for countries receiving aid than for those not. That, of course, is not a surprise. The study expects, or at least hopes, that aid will be directed to poorer countries. However, the results imply that unconditional on these time-invariant factors, aid has not had such an effect, while our other parameters are highly significant. There remains much to learn as to how aid, policy, and growth are related.

This study has used panel data to examine poverty, inequality, and economic growth in the MENA region. Further, it also examines the impact of foreign aid and remittances as the percentage share of GDP on poverty reduction and the poverty gap. It has four broad findings. First, the study reveals that the MENA countries have unusually low poverty rates compared to the developing world. Only 3.5% of the MENA region population live below the international poverty standard of $1.90 per person per day in 2010–2019. These enviable statistics related to poverty reduction are commendable; however, the more interesting observation of the MENA region is maintaining such low poverty rates even in times of sluggish economic growth. Achieving these poverty levels can be attributed mainly to the rapid income growth in the region prior to 1985. However, MENA has managed to prevent a sizeable increase in poverty despite its low economic growth. Second, the MENA region has become similar to other developing regions in the last 20 years regarding income distribution. Altogether, the region has had an increasing share of income among the bottom quintile of the population. Using panel data analysis, it is observed that high-income countries are more efficient in reducing poverty than low-income countries. This indicates that the countries with low-income inequality are better at poverty reduction than countries with high-income inequality [72]. This helps in explaining the sustenance of low poverty rates in the MENA region despite having negative per capita GDP growth throughout the last two decades. Due to initial low levels of income inequality due to the oil prices boom, some MENA regions do not require high economic growth rates to keep a low incidence of poverty. Third, this study has studied two key reasons, namely remittances as a percentage of GDP and foreign aid, to contribute to poverty reduction and low-income inequality for MENA. The panel data estimates indicate that foreign aid and international remittances negatively impact poverty in the MENA region. A 10 percentage points increase in the total GDP’s remittance share reduces the poverty headcount ($1.90 per person/day) by 8.3% on average. A 10-percentage point rise in the inflow of foreign aid reduces poverty headcount by 1.02% for foreign aid. Further, we have applied the system-GMM technique for robustness and found similar results. Thus, this shows that remittances and foreign aid have helped the MENA region maintain low poverty levels and income inequality despite having low economic growth.

However, the present study is limited in its scope and does not reveal the future aspects of relationships among poverty, inequality, and growth for the MENA region. The question remains whether the MENA region would maintain low poverty levels in the absence of accelerated economic growth. The likelihood of this happening is very low because of multiple factors. The relationship between poverty as a response to income growth and its distribution is the same for other MENA economies. The underprivileged or needy will be benefited only in terms of a rise in their income when the share of income continues to rise for the poorest quantile in general.

Moreover, the increase in income for the poor in MENA is significantly driven by the remittances and foreign aid received, as evidenced by the present study’s findings. As the competition increases from the other labor exporting countries in both the region’s foremost labor destinations, namely the European Region and the Arab Gulf region, and rising barriers, the remittances are getting affected, which creates a problem for the poor. Simultaneously, the fiscal constraints faced by MENA governments and the pressure to privatize are making it difficult for the regional countries to provide public employment.

However, a modest increase in economic growth is expected to substantially reduce poverty incidence due to the relatively equal income distributions in the region. Therefore, policymakers’ challenge is to determine methodologies to speed up growth while maintaining the maximum extent possible the distributional benefits achieved by the poor in the last 30 years of the MENA region.

## Supporting information

S1 Data (XLS)

S1 Appendix (DOCX)
