# Supplementary material for: How foreign aid and remittances affect poverty in MENA countries?
Source: PLoS One. 2022 Jan 19;17(1):e0261510. doi: 10.1371/journal.pone.0261510 (PMC8769347; doi:10.1371/journal.pone.0261510)
Supplement: S1 Appendix — (DOCX) [file pone.0261510.s002.docx]

**APPENDIX**

**Table A1: Elasticity of Poverty concerning Aid and Remittances (Fixed Effect)**

|  | Dependent variable = poverty headcount at $1.90/person/day | | | | | Dependent variable = poverty gap at $1.90/person/day | | | | |
| --- | --- | --- | --- | --- | --- | --- | --- | --- | --- | --- |
|  | (1) | (2) | (3) | (4) | (5) | (6) | (7) | (8) | (9) | (10) |
| ln GDP  per capita | -11.84*** | -10.86*** | -17.41*** | -19.58*** | -19.45*** | -4.514*** | -4.005*** | -6.977*** | -7.557*** | -7.515*** |
|  | (-38.82) | (-32.09) | (-32.43) | (-41.04) | (-41.05) | (-31.20) | (-25.10) | (-26.96) | (-31.46) | (-31.32) |
| Gini Index |  | 0.109** | 0.426*** | 0.306*** | 0.283*** |  | 0.0875*** | 0.237*** | 0.187*** | 0.180*** |
|  |  | (2.82) | (9.05) | (7.73) | (7.15) |  | (4.80) | (10.46) | (9.42) | (8.97) |
| ln net aid  received |  |  | -0.0969 | -0.0503 | -0.0299 |  |  | -0.0406 | -0.0193 | -0.0123 |
|  |  |  | (-1.02) | (-0.65) | (-0.39) |  |  | (-0.88) | (-0.49) | (-0.32) |
| Remittances |  |  |  | -0.835*** | -0.830*** |  |  |  | -0.308*** | -0.306*** |
|  |  |  |  | (-16.91) | (-16.97) |  |  |  | (-12.39) | (-12.37) |
| MENA  excluding HIC |  |  |  |  | -5.695*** |  |  |  |  | -1.928* |
|  |  |  |  |  | (-3.71) |  |  |  |  | (-2.48) |
| Constant | 128.7*** | 114.1*** | 163.6*** | 193.1*** | 192.8*** | 48.94*** | 40.27*** | 62.33*** | 71.22*** | 71.12*** |
|  | (41.79) | (27.57) | (27.42) | (35.82) | (36.09) | (33.47) | (20.63) | (21.68) | (26.24) | (26.30) |
| R-square | 0.565 | 0.541 | 0.635 | 0.739 | 0.746 | 0.459 | 0.442 | 0.557 | 0.635 | 0.639 |
| sigma_u | 5.050 | 4.607 | 2.975 | 3.614 | 2.579 | 2.201 | 1.992 | 1.314 | 1.584 | 1.185 |
| sigma_e | 10.604 | 10.613 | 11.116 | 9.065 | 8.981 | 5.033 | 5.007 | 5.358 | 4.564 | 4.547 |
| rho | 0.185 | 0.158 | 0.067 | 0.137 | 0.076 | 0.160 | 0.137 | 0.0567 | 0.107 | 0.064 |
| Observations | 1260 | 1260 | 1260 | 1260 | 780 | 1260 | 1260 | 1260 | 1260 | 780 |

Note: *Significance level of the difference: * p<0.05, ** p<0.01, *** p<0.001*

Source: *Authors' calculations, using data from World Development Indicators*

*Robust standard errors clustered at the country-level in parentheses.*

**Table A2: Hausman Test**

|  | Coefficients | |  |  |
| --- | --- | --- | --- | --- |
|  | (b) | (B) | (b-B) | sqrt(diag(V_b-V_B)) |
|  | fixed | random | Difference | S.E. |
| ln GDP per capita, PPP | -19.45 | -19.49 | 0.04 | 0.142 |
| Gini Index | 0.28 | 0.28 | -0.00 | 0.010 |
| ln net aid received | -0.02 | -0.03 | 0.00 | 0.017 |
| Remittances | -0.83 | -0.83 | 0.00 | 0.010 |
| MENA excluding HIC | -5.69 | -5.77 | 0.08 | 0.776 |
| chi2(5) | 0.480 |  |  |  |
| Prob>chi2 | 0.993 |  |  |  |

Source: *Authors' calculations, using data from World Development Indicators.*

*Note: b = consistent under H0 and Ha*

*B = inconsistent under Ha, efficient under H0*

*H0: difference in coefficients is not systematic*

*Chi2 (5) = (b-B) ‘ [(V_b-V_B) ^ (-1)] (b-B) = 0.48*

*Prob>chi2 = 0.9930*
